# Diagnosis and management of complications from the treatment of primary central nervous system tumors in adults

**DOI:** 10.1093/neuonc/noad038

**Published:** 2023-02-27

**Authors:** Michael Weller, Emilie Le Rhun, Martin Van den Bent, Susan M Chang, Timothy F Cloughesy, Roland Goldbrunner, Yong-Kil Hong, Rakesh Jalali, Michael D Jenkinson, Giuseppe Minniti, Motoo Nagane, Evangelia Razis, Patrick Roth, Roberta Rudà, Ghazaleh Tabatabai, Patrick Y Wen, Susan C Short, Matthias Preusser

**Affiliations:** Department of Neurology, University Hospital and University of Zurich, Zurich, Switzerland; Department of Neurology, University Hospital and University of Zurich, Zurich, Switzerland; Department of Neurosurgery, Clinical Neuroscience Center, University Hospital and University of Zurich, Zurich, Switzerland; The Brain Tumour Center at the Erasmus MC Cancer Institute, Rotterdam, The Netherlands; Department of Neurological Surgery, University of California, San Francisco, California, USA; Department of Neurology, David Geffen School of Medicine at UCLA, Los Angeles, California, USA; Center of Neurosurgery, Department of General Neurosurgery, University of Cologne, Cologne, Germany; Brain Tumor Center, Hallym University Sacred Heart Hospital, Anyang, South Korea; Neuro Oncology Cancer Management Team, Apollo Proton Cancer Centre, Chennai, India; Department of Neurosurgery, The Walton Centre NHS Foundation Trust & University of Liverpool, Liverpool, UK; Department of Medicine, Surgery and Neurosciences, University of Siena, Policlinico Le Scotte, Siena, Italy; IRCCS Neuromed, Pozzilli, IS, Italy; Department of Neurosurgery, Kyorin University Faculty of Medicine, Tokyo, Japan; Third Department of Medical Oncology, Hygeia Hospital, Marousi, Athens, Greece; Department of Neurology, University Hospital and University of Zurich, Zurich, Switzerland; Division of Neuro-Oncology, Department of Neuroscience, City of Health and Science and University of Turin, Turin, Italy; Department of Neurology & Neuro-Oncology, Hertie Institute for Clinical Brain Research, Center for Neurooncology, Comprehensive Cancer Center, German Cancer Consortium (DKTK), Partner site Tübingen, University Hospital Tübingen, Eberhard Karls University Tübingen, Tübingen, Germany; Center for Neuro-oncology, Dana-Farber Cancer Institute and Harvard Medical School, Boston, Massachusetts, USA; Leeds Institute of Medical Research, University of Leeds, Leeds, UK; Department of Clinical Oncology, Leeds Teaching Hospitals NHS Trust, Leeds, UK; Division of Oncology, Department of Medicine 1, Medical University, Vienna, Austria

**Keywords:** adverse affects, dose, interruptions, prevention, reexposure, toxicity

## Abstract

Central nervous system (CNS) tumor patients commonly undergo multimodality treatment in the course of their disease. Adverse effects and complications from these interventions have not been systematically studied, but pose significant challenges in clinical practice and impact function and quality of life, especially in the management of long-term brain tumor survivors. Here, the European Association of Neuro-Oncology (EANO) has developed recommendations to prevent, diagnose, and manage adverse effects and complications in the adult primary brain CNS tumor (except lymphomas) patient population with a specific focus on surgery, radiotherapy, and pharmacotherapy. Specifically, we also provide recommendations for dose adaptations, interruptions, and reexposure for pharmacotherapy that may serve as a reference for the management of standard of care in clinical trials. We also summarize which interventions are unnecessary, inactive or contraindicated. This consensus paper should serve as a reference for the conduct of standard therapy within and outside of clinical trials.

The therapeutic repertoire of interventions for patients with primary brain tumors is steadily increasing. New interventions are integrated into current standards of care and interactions between novel and standard treatments are often insufficiently studied. Furthermore, how to diagnose and manage complications from cancer treatment is an evolving field. Toxicity in the context of clinical trials is typically captured using Common Terminology Criteria for Adverse Events (CTCAE).^1^ However, there is a paucity of data and consensus on how to prevent adverse events and manage cancer patients once such complications are noted. Therefore, the Guideline Committee of the European Association of Neuro-Oncology (EANO) selected a task force from EANO to develop recommendations for clinical practice. These recommendations are restricted to approved or commonly used treatment approaches for primary brain tumors in adults with the exception of primary central nervous system (CNS) lymphoma. Novel systemic treatments for extra-CNS cancers are only covered if they have direct implications for neuro-oncology care. Complications from the brain tumors themselves have been addressed elsewhere.^2^ Given the broad significance of the topic, the EANO leadership invited colleagues from the United States and the Asia Pacific region to review and help develop this guideline, too. Consensus was achieved by several rounds of review and direct personal discussions in some instances. There was no formal voting process on the recommendations.

## Surgery

### General

Complications from neurosurgery are inconsistently reported in the neurosurgical literature,^3^ although standardization of reporting of surgical complications can improve the safety and quality of treatment.^4^ The Landriel Ibañez Classification was developed in 2011 to standardize reporting and classification of surgical and medical complications in patients undergoing neurosurgery.^5^ Neurosurgical complications are graded from I to IV according to severity (Supplementary Table S1) and can be readily applied to neurosurgical oncology where they maybe more frequently observed than for other brain surgeries.^6^ In a cohort of 362 patients operated for a brain tumor, 27 patients (7.5%) experienced an unplanned rehospitalization within 30 days after surgery, mainly due to neurological, infection and venous thromboembolic complications. Nineteen readmissions could have been prevented.^3^ All surgical complications in brain tumor patients have management implications, for example, the start of radiotherapy and chemotherapy will be delayed in patients with glioblastoma who have a bone flap infection requiring surgical removal and several weeks of intravenous antibiotics. Furthermore, the prognosis may be adversely affected—particularly if surgical complications result in a deterioration in performance status and neurological function such that the patient is no longer eligible for oncological treatment or participation in clinical trials. Prevention, prophylaxis, and minimization of surgical complications are therefore an essential part of patient care. Standard operating procedures and the center’s experience with brain tumor surgery may help to reduce the rate of complications and lead to more favorable early postsurgical outcomes.^7,8^ MRI including diffusion-weighted imaging (DWI) sequences should be obtained within 48–72 hours after surgery not only to assess extent of resection, but also to detect local complications such as perioperative ischemia. Patients should be seen at least once after surgery by the operating surgeon in an interval of 2–4 months.

### Venous Thromboembolic Events

Venous thromboembolic events are common in brain tumor patients, but not primarily a complication of therapeutic interventions, except of surgery. Their prophylaxis and their management are detailed in another EANO ESMO guideline.^2^ Sinus venous thrombosis in the brain is an exception because that might directly relate to neurosurgical interventions.

### Ischemia

Cerebrovascular accidents are important complications of neurosurgical interventions. The majority of these ischemic events are asymptomatic. The rate of ischemia varies markedly in the literature and depending on the primary tumor type. In a series of 82 “low grade” gliomas, 19 patients (23%) had radiological evidence of ischemia after surgery.^9^ In a cohort of 245 patients operated for newly diagnosed glioblastoma, 40 patients (16%) had postoperative ischemia, 119 patients (49%) had no ischemia, and 80 patients (33%) had unspecific DWI lesions on an MRI performed within 72 after surgery with T1-weighted (T1-w) sequences before and after intravenous administration of contrast, T2-weighted sequences and DWI sequences with calculated ADC maps.^10^ A higher incidence of new ischemic lesions was noted in patients operated for a recurrent glioma (*n* = 20, 80%) as compared with patients with surgery for newly diagnosed glioma (*n* = 26, 31%), and both new permanent and transient neurological deficits were more frequently noted after surgery for recurrent glioma.^11^

Early cerebrovascular complications arise from direct arterial or venous injury during surgery. Meticulous attention to vascular anatomy, main vessels, and perforators, and preservation of venous drainage can reduce the risk, however, in higher-grade gliomas blood vessels are more fragile and tortuous, and in meningioma the tumor may completely encase arteries and veins risking vessel injury during resection. Other risk factors include potential coexisting medical diseases, such as atrial fibrillation where anticoagulants are stopped preoperatively. Treatment of peri- and postoperative ischemia is with neuro-rehabilitation and there is no role for thrombolysis in patients with intra-axial malignant tumors due to the risk of intracranial hemorrhage.^12^ A neurovascular work-up may be indicated. The risk of ischemic stroke due to embolic occlusion of the middle cerebral artery in the perioperative period is very low—however, in the rare circumstance where this occurs, mechanical intraarterial thrombectomy may be considered.^13,14^

Regarding initiation of further therapy, it is important to differentiate intraoperative ischemia from hypercoagulable postoperative stroke or embolic stroke which requires a regular stroke work-up. Moreover, surgical devascularization ischemia should not be an exclusion criterium for clinical trials whereas hypercoagulable or embolic stroke might be.

### Hemorrhage

Hemorrhage is often reported on postoperative CT or MRI and affects 1.1%–4.4% of brain tumor patients.^15^ It often remains asymptomatic. Postoperative hemorrhage after biopsies has been noted in 53 of 186 patients (28%) with glioblastoma (volume >0.05 cm^3^ in 17 cases), in 19 of 76 patients (25%) with astrocytoma, and in 7 of 25 patients (28%) with oligodendroglioma.^16^ Meticulous intraoperative hemostasis will minimize the risk of postoperative hemorrhage. Various surgical techniques are available including bipolar cautery, irrigation, and hemostat agents, for example, oxidized cellulose, gelatin matrix, and thrombin. Hemorrhage requiring repeat surgery is more likely to occur in patients with underlying clotting disorders or those with poorly controlled hypertension and those with thrombocytopenia which should be ascertained before surgery.^17^ Guidelines on the management of antithrombotic and anticoagulant therapy in patients receiving these treatments prior to surgery for a brain tumor are detailed in Supplementary Table S2. Perioperative hemorrhage unrelated to a coagulation disorder does not constitute a contraindication for the initiation of further treatment such as radiotherapy and chemotherapy.

### Peri- and Postoperative Seizures

Intra- and postoperative seizures can occur during surgery using direct cortical stimulation in eloquently located tumors, particularly during awake surgery without seizure protection by general anesthesia. The incidence of intraoperative seizures in awake surgery ranges from 3.4% to 7.3%.^18,19^ The practice of postoperative seizure prophylaxis varies widely among physicians, with markedgeographic variation.^20^ The SNO/EANO practice guideline does not recommend prescribing antiepileptic drugs as primary prophylaxis in the peri- or postoperative situation, even for awake surgery.^21^ There are no reliable data on long-term seizures after surgery since the cause of epilepsy might be multifactorial.

### Wound Healing

Surgical wounds should be healed by 2 weeks following which patients can start oncological treatments including radiotherapy, chemotherapy, and immunotherapy. Bevacizumab should be stopped at least 4 weeks prior to any planned repeat craniotomy to reduce wound healing problems.^22^ Similarly, the first dose of bevacizumab should be delayed for 4 weeks postoperatively even after biopsy only and should only be given once the wound has healed.^23^ Patients and caregivers should be informed about wound care and symptoms and signs of impaired wound healing.

### Infections

Postoperative infections can be categorized as superficial wound infection, bone flap infection, intra-parenchymal abscess, meningitis, and ventriculitis. The overall postoperative infection rate in glioma surgery is 0.5%–5%^15,24^ and recognized risk factors include diabetes, corticosteroids, and smoking. The risk of infection is not increased by the use of 5-aminolevulinic acid, ultrasound, awake surgery, or electrophysiology monitoring in patients operated for brain tumors.^25^ Despite anecdotal evidence, postoperative bacterial infection does not confer a survival benefit in glioblastoma.^26^

Bundles of care are often implemented to reduce infection rates, for example, pre- and postoperative medicated hair wash, use of combined chlorhexidine and betadine skin preparation and administration of perioperative antibiotics. Definitive evidence to support their use remains conflicting and surgeon and hospital variation exist in practice.^27–29^ As a minimum, perioperative antibiotics, careful skin preparation, and meticulous attention to aseptic technique by the surgical team are recommended.

Bone flap infections should be managed with bone flap removal and appropriate intravenous antibiotics according to microbiology culture results. If a ventriculoperitoneal shunt or an intraventricular access device for intrathecal chemotherapy becomes infected, this should be managed with surgical removal of the hardware and appropriate intravenous antibiotics according to microbiology culture results. Appropriate aseptic technique and experienced teams are recommended for the management of such devices.

### Hydrocephalus

Postoperative hydrocephalus affects up to 7% of patients undergoing glioma resection^30^ and 2.1% of the patients after posterior fossa surgery.^31^ Acute hydrocephalus should be managed with emergency external ventricular drain and cerebrospinal fluid (CSF) analysis to ensure there is not coexisting infection. Once CSF is confirmed as sterile and there is confirmed persistent hydrocephalus clinically and on imaging, patients should undergo insertion of a ventriculoperitoneal shunt. Use of antibiotic-impregnated catheters reduces the risk of shunt infection to ~2%.^32^ Valve choice is at the discretion of the surgeon.

### Main Expert Recommendations

Brain tumor surgery should be performed in specialized centers.^7,8^
Risk factors for infection, ischemia, and bleeding should be identified and considered prior to surgery.
DWI sequences should be included in the postoperative MRI obtained within 24–72 hours after surgery.
Postoperative infections require vigorous treatment per se to avoid delays of further oncological therapies.
Patients and caregivers should be informed about wound care and symptoms and signs potentially associated with complications.
Radiotherapy and systemic antitumor therapy should not be started unless the patient has recovered from postoperative complications and is in a stable condition.
The indication for neurorehabilitation should be routinely assessed after brain tumor surgery.^8^

## Radiotherapy

Radiotherapy for patients with brain tumors should be administered at specialized centers. In most patients with primary brain tumors, the target volume of radiotherapy is the tumor with a safety margin, but in certain situations such as widespread disease or for certain entities such as medulloblastoma, whole brain radiotherapy (WBRT), or craniospinal radiotherapy, respectively, are indicated. Treatment plans should adhere to international and national guidelines as applicable. Current advanced radiotherapy techniques including image guidance, intensity-modulated, arc delivered photons and protons improve dose conformality and ensure precise dose delivery to the tumor, while limiting exposure of surrounding normal tissues and organs at risk. Nevertheless, significant acute and late treatment-related neurological toxicity can develop, especially in patients receiving high-dose radiotherapy for large volumes or radiotherapy in combination with some systemic therapies.

### Acute Toxicities

For patients receiving conventionally fractionated radiotherapy, typically 54–60 Gy in 27–33 fractions are prescribed to partial brain for most types of primary brain tumors. Acute toxicities are defined as those occurring during or within a few weeks, by convention <90 days from the commencement of radiotherapy include hair loss, skin changes (erythema and desquamation), and fatigue. Some patients also experience headache, nausea, vomiting, and signs of raised intracranial pressure from increased edema, as well as worsening of preexisting focal neurologic deficits and seizure. These acute radiation adverse events are usually transient.^33^

Transient systemic dexamethasone may be needed to control symptoms of raised intracranial pressure but systematic prophylactic steroids should not be used. The minimal dose of steroids guided by the clinical neurological evaluation should be used. Radiation dermatitis is characterized by a variety of skin changes, including edema, erythema, and dyspigmentation. Skin necrosis may rarely occur after high doses of irradiation or in individuals with radiosensitivity syndromes. Although no standard criteria for its management exists, washing with only mild soap and water and the use of topical corticosteroids have been recommended for radiation dermatitis.^34^ Skin toxicity of RT may increase in patients who receive concurrent systemic treatments but this does not include agents commonly administered to patients with primary brain tumors, except for combined BRAF/MEK inhibition. According to the Consensus Guidelines from the Eastern Cooperative Oncology Group, BRAF inhibitors and/or MEK inhibitors should be stopped ≥3 days before and until after fractionated RT and ≥1 day before and after stereotactic radiosurgery (SRS).^35^ It remains controversial whether coexposure to older anticonvulsants such as phenytoin and carbamazepine enhances the risk of allergic reactions in patients undergoing RT,^36^ but this question has lost clinical relevance with the introduction of several novel anticonvulsants that are preferred over the old drugs for multiple reasons.^2^

An increasing number of patients undergoing RT have cardiac implantable electronic devices (CIED), cardiac pacemakers, and implanted cardioverters/defibrillators. Ionizing radiation, especially thoracic RT (as for craniospinal irradiation), can damage a CIED. Therefore, a collaborative multidisciplinary approach is necessary to provide effective and safe RT for patients with CIED. In general, CIED should never be located in the beam, and the beam energy should be limited to 6–10 MV, with the cumulative scatter radiation dose limited to 2 Gy. After the course of RT, CIED evaluation should be performed 1, 3, and 6 months due to the risk of latent damage.^37^

RT may need to be interrupted transiently in case of intercurrent disease, compromise in patients’ physical or neurological condition, or significant treatment toxicity. Causes of interruption include increased edema leading to neurological symptoms, severe fatigue or uncontrolled nausea and vomiting, especially during concurrent administration of chemotherapy and RT. Every effort must be made to recognize early reactions and to ensure that they are actively managed (steroids, antiemetics including 5-HT_3_-receptor antagonists like ondansetron or granisetron, prochlorperazine, and metoclopramide^38^) to reduce the risk of treatment delay. Bevacizumab (see below) can mitigate RT-associated neurotoxicity and enable treatment to be completed as planned.

Further risks for treatment interruption during RT include the development of neutropenia or thrombocytopenia (or both), especially in the presence of concurrent administration of chemotherapy and increasing volumes of active bone marrow in the radiation field, for example in spinal fields. The need for and frequency of blood value monitoring during RT depends on concurrent treatment, notably cancer chemotherapy, and comorbidities. Grade 3 or 4 hematological toxicity is rarely recorded during RT for small brain volumes, although it occurs in up to 10% of adult patients during craniospinal irradiation. A transient interruption of treatment of 3–5 days, reduction of radiation total dose or daily fraction size, and interruption of chemotherapy should be considered in patients with grade 3 or 4 hematological toxicity or febrile neutropenia. It should be noted that for patients receiving RT and concomitant chemotherapy the use of hematopoietic growth factors should be avoided because of the increased risk of complications and death.^39,40^

In patients with fast-growing tumors, such as glioblastoma and medulloblastoma, transient treatment interruption should be compensated by increasing the total dose and/or dose per fraction using radiobiological-based calculations.^41^

The management of longer interruptions requires medical decisions to be taken on a case-by-case basis. For patients with rapid worsening of neurological condition due to cancer progression causing severe functional disability or inacceptable treatment toxicity, a premature discontinuation of treatment should be considered.

### Late, Commonly Irreversible Toxicities

These toxicities occur several months and by definition more than 90 days after treatment and include radionecrosis, cognitive impairment as well as permanent hair loss, hypopituitarism and cataracts.^33,42^ Pseudoprogression is commonly defined as an increase of lesion volume of 25% or more that resolves without institution of a new anticancer treatment except steroids in the absence of a new lesion whereas radionecrosis is defined as the appearance of necrosis within the treated target volume irrespective of size. Depending on the volume and site, these events can be either asymptomatic or symptomatic. In the AVAglio study, pseudoprogression was observed in 43 of 463 patients (9.3%) treated with standard of care versus 10 of 458 patients (2.2%) of patients treated with bevacizumab in addition to standard of care.^43^ The initial observation of an association between pseudoprogression and *MGMT* promoter methylation^44^ was not confirmed in the AVAglio study.^45^ Radionecrosis is a rare complication of brain radiotherapy which can develop months to years after radiotherapy. On MRI it appears as increased T2 signal with T1 enhancement, often described as “soap bubble” in character. Overall, severe toxicity (CTCAE grade 3 or more) is seen in less than 3% of patients when using doses of 50–60 Gy given in 1.8–2.0 Gy per fraction.^46^ In contrast, in the setting of reirradiation, the risk of symptomatic radionecrosis ranges from 5% to 20% depending on dose and irradiated volumes.^46,47^ Hypofractionated radiation schedules with total doses of 24–40 Gy given in 3–15 fractions may reduce the risk of brain necrosis associated with single-fraction SRS when treating large (>3 cm) brain lesions or in the setting of reirradiation.^47^

Symptomatic patients with radionecrosis are usually treated with steroids or bevacizumab,^48,49^ whereas surgery is indicated if suspicion of tumor progression remains or if symptomatic relief of mass effect is desirable.

Neurocognitive changes are well-described potential late effects of brain irradiation which occur months to years after treatment in up to 50%–90% of adult brain tumor patients who survive more than 6 months postirradiation, depending on radiation dose and fractionation and treated volumes.^50^ The incidence and magnitude may be higher in young adults and several strategies are employed to minimize this complication including avoidance or delay of RT in low-grade tumors, reducing the dose in molecularly favorable tumors and employing high-precision RT techniques.^51^ The majority of patients treated with WBRT develop deterioration in cognitive function and quality of life,^52^ although the incidence can be also significant in the long-term follow-up of patients receiving partial brain RT for a brain tumor^53,54^ Limitation of radiation dose to the bilateral hippocampal dentate gyri (hippocampal avoidance) during WBRT may ameliorate neurocognitive decline.^55,56^ In young adults with lower-grade gliomas and craniopharyngiomas treated to a dose of 54 Gy in 30 fractions at 1.8 Gy per fraction, a mean dose <30 Gy to left hippocampus as dose constraint prevented neurocognitive decline.^57^ Modern conformal RT techniques, both photon intensity-modulated RT and protons (intensity-modulated proton therapy), should be considered to achieve these constraints in young adults with low grade or benign tumors with expected long-term survival.^57,58^

Proton radiotherapy may also reduce late toxicity by limiting dose to normal brain, although it has not been shown to delay time to cognitive failure in glioblastoma.^59^ There is also growing interest in proton therapy for minimizing the incidence of RT-induced lymphopenia which may potentially impact outcome.^60,61^

Memantine intake during WBRT or craniospinal therapy is not recommended considering the absence of convincing data for a protective effect in patients with brain metastases.^62^ Hypopituitarism is a common, dose-dependent late effect when the pituitary dose exceeds 30 Gy and individual hormone deficiencies occur at lower doses, so early identification of deficits to permit replacement therapy is important.^29^ Follow-up should therefore include regular neurological and endocrinological follow-up and access to appropriate rehabilitation.^63^

Patients with good prognosis tumors should also be made aware of rarer long-term late effects including increased risk of vascular damage and stroke, especially in patients with tumors close to the central arterial circulation.^64^ A rare but important complication to recognize and differentiate from recurrence and stroke is the usually self-limiting stroke-like migraine syndrome (SMART).^65^ There is also a low (around 2% at 20 years) risk of radiation-induced second malignancies in the decades after completing radiotherapy.^66^

### Main Expert Recommendations

Radiotherapy for patients with brain tumors should be offered only by specialized centers.
Patient age, tumor location, and prognosis should determine the choice of dose, fractionation regimens, and volume.
Prophylactic systemic steroids should not be used when initiating radiotherapy.^2^
Low-to-moderate doses of dexamethasone (≤8 mg) given in 1 or 2 daily doses should be considered in case of symptomatic brain edema or neurological deterioration during radiotherapy.^2^
Transient interruption of radiotherapy may be necessary in case of intercurrent disease with or without neurological deterioration, and acute treatment toxicity should be actively managed to avoid treatment interruptions.
Permanent discontinuation of radiotherapy should be considered only for patients with severe functional disability or unacceptable treatment toxicity.
Prevention of late adverse events by choice of total dose, dose distribution, and beam modality remains a priority because late sequelae of radiotherapy are essentially irreversible.

## Tumor-Treating Fields

Tumor-Treating Fields (TTFields) are a noninvasive locoregional treatment modality delivering alternating electric fields with 200 kHz by a device.^67^ Transducer arrays are localized directly on the shaved scalp of glioblastoma patients to cover the tumor bed. TTFields are contraindicated in patients with skull defects, bullet fragments, and allergy to hydrogels. The most common TTFields-related adverse events are locoregional dermatologic adverse events,^68^ that is, dermatologic side effects beneath the transducer arrays. In a systematic review of the literature, data on safety were collected among 12 studies including 11 558 patients. The main adverse events were skin adverse events with a pooled prevalence of 38.4%, mostly mild to moderate. Headaches were reported in 5 studies, with a pooled prevalence of 21.7%. An overview of action items for the management of dermatological toxicity is provided in Supplementary Table S3. Systemic adverse events of TTFields have not been observed. In a cohort of 156 patients with ventriculoperitoneal shunt, 6 patients experienced 7 instances of TTFields therapy-related skin toxicity at the shunt site or the resection scar, but TTFields did not interfere with ventriculoperitoneal shunt function.^69^ Datasets for the safety in association with pacemakers^70^ or defibrillators^71^ only contain very small sample sizes, remain inconclusive, and need validation in prospective cohorts.

### Main Expert Recommendations

The application of TTFields with active implanted medical devices needs thorough evaluation of potential interactions. In case of simultaneous application, careful monitoring strategies are warranted.
TTFields should be transiently interrupted for any type of dermatologic adverse events.
TTFields should be discontinued for dermatologic ulcerations grade 3 or any indication of interference with active implanted medical devices.

## Cancer Pharmacotherapy

### General

The profile of tolerance and complications of cancer pharmacotherapy for primary brain tumors differ from those used in general oncology, but some practical considerations apply (Note S1). Mode of administration and the minimum dataset of laboratory values to be considered at treatment initiation and during follow-up are provided in Table 1.

**Table 1.** Mode of Administration and Laboratory Values Required to Allow Treatment Initiation (Expert Opinion)

	Dosing	Mode of Administration	Preconditions for Drug Administration						Prophylaxis	Controls
			Neutrophils	Lymphocytes	Platelets	Hemoglobin	Liver Enzymes	Others		
Temozolomide	75 mg/m^2^ during radiotherapy 7 days a week 150–200 mg/m^2^ (days 1–5 out of 28 days) as a single agent; Minimal dose during maintenance: 100 mg/m^2^	Oral, i.v. application no longer available Capsules should be swallowed whole with approximately 250 mL (1 glass) of water while fasting, in the morning, ie, at least 1 h before a meal or 2 h after a meal Concomitant with RT, capsules should be taken approximately 1 h before administration of RT	≥1.5 × 10^9^/L	—	≥100 × 10^9^/L	—	Normal liver function	Nonhematological toxicity CTCAE grade ≤1, except for alopecia, nausea, and vomiting	*Antiemetic prophylaxis* *Concomitant:* 5-HT_3_ antagonists usually only required for the initial 2–3 doses of temozolomide. Then simple antiemetic prophylactic treatment with metoclopramide or domperidone is sufficient for most patients, some patients may not require any antiemetic *Maintenance:* Strongly recommended prophylaxis: 5-HT_3_ antagonist, low dose, eg, ondansetron 4 mg or granisetron 1 mg p.o. 1 h prior to temozolomide administration *Pneumocystis jirovecii prophylaxis* *Concomitant:* Per label required for all patients regardless of lymphocyte count during RT and beyond until any lymphopenia has recovered to CTCAE grade ≤1, but debatable for patients without corticosteroids and without lymphopenia *Maintenance:* all patients if lymphocytes ≤0.8 × 10^9^/L (CTCAE grade ≥1) or CD4 count ≤ 200/μL, particularly patients receiving steroids should be observed closely for the development of *pneumocystis jirovecii* pneumonia *Proposed prophylaxis* Pentamidine inhalations by nebulizer once a month or trimethoprim–sulfamethoxazole (Bactrim forte) 1 tablet/3× per week	During concomitant radiochemotherapy: weekly complete blood count and liver enzymes During maintenance: complete blood count (days 21 and 28) and, liver enzymes at day 21
Procarbazine	100–150 mg daily, in the PCV protocol 60 mg/m^2^ on days 8 through 21	Oral	≥1.5 × 10^9^/L	—	≥100 × 10^9^/L	—	—	Nonhematological toxicity grade ≤1, except for alopecia, nausea, and vomiting	5-HT_3_ antagonist, low dose, eg, ondansetron 4 mg or granisetron 1–2 mg p.o. 1 h prior to administration	
Lomustine	130 mg/m^2^ single agent, 110 mg/m^2^, in the PCV protocol or in combination with bevacizumab, commonly capped at 200 mg, ×6–8 weeks	Oral	≥1.5 × 10^9^/L	—	≥100 × 10^9^/L	—	—	Nonhematological toxicity grade ≤1, except for alopecia, nausea and vomiting Pulmonary examination and nerve conduction studies prior to first cycle not routinely, but only with appropriate history	Recommended prophylaxis: 5-HT_3_ antagonist or metoclopramide p.o. 1 h prior to lomustine administration	At days 28 and 35 complete blood counts, at day 42 complete blood count and blood chemistry
Vincristine	2 mg, days 8 and 29 in the PCV protocol	i.v.	≥1.5 × 10^9^/L	—	≥100 × 10^9^/L	—	—	Discontinuation for any clinical signs of peripheral neuropathy		Complete blood count, vincristine alone is only weakly myelosuppressive, but commonly given together with other other myelosuppressive agents
PCV		Oral and i.v.	≥1.5 × 10^9^/L	—	≥100 × 10^9^/L	—	—	See lomustine, procarbazine, and vincristine Pulmonary examination and nerve conduction studies prior to first cycle not routinely, but only with appropriate history	Recommended prophylaxis: 5-HT_3_ antagonist or metoclopramide p.o. 1 h prior to lomustine administration, 5-HT_3_ antagonist on day 1 and 2 p.o. 1 h prior procarbazine and metoclopramide as needed during procarbazine administration	At days 8 and 29 complete blood counts, after week 6 complete blood count and blood chemistry
Bevacizumab	10 mg/kg × 14 days or 15 mg/kg × 3 weeks i.v.	i.v.	≥1.5 × 10^9^/L	—	≥100 × 10^9^/L	—	—	Liver function: CTCAE grade 1 allowed Microproteinuria allowed	—	Complete blood count and liver enzymes every 14 days and additional urine status 48 h before next infusion cycles Weekly or even shorter controls might be necessary depending on laboratory values Blood pressure prior/during and after infusion Careful assessment for any clinical signs of deep vein thrombosis Educate patients to report immediately any signs of shortness of breath (pulmonary embolism or myocardial infarction) or abdominal pain
Vemurafenib	960 mg twice daily	Oral Avoid concomitant administration with strong CYP3A4 inhibitors or inducers	≥1.5 × 10^9^/L	—	≥75 × 10^9^/L	—	—	ECG (QT time) Dermatological examination prior to drug initiation	Avoid sun exposure, use of sunscreen, not to be used during RT	Every 4 weeks blood counts and blood chemistry including electrolytes, First 3 months monthly ECG (QT time), thereafter every 3 months Dermatological examination every 3 months
Dabrafenib/tramatenib	Dabrafenib 2 mg daily, trametinib 150 mg twice daily	Oral Take dabrafenib and trametinib with water at least 1 hour before or 2 hours after a meal Avoid concurrent administration of strong inhibitors of CYP3A4 or CYP2C8	≥1.5 × 10^9^/L	—	≥75 × 10^9^/L			Cardiac function: ECG, left ventricular ejection fraction Dermatological examination prior to drug initiation	Avoid sun exposure, use of sunscreen, not to be used during RT	Every 4 weeks blood counts and blood chemistry including electrolytes Monthly control of blood pressure Every 3 months left ventricular ejection fraction Dermatological examination every 3 months
Laroctrectinib and entrectinib	Larotrectinib: 100 mg twice daily; minimal dose: 50 mg twice daily Symptoms of overdose are not established. In the event of overdose, physicians should follow general supportive measures and treat symptomatically Entrectinib: 600 mg once daily; minimal dose: 200 mg once daily	*Larotrectinib* Oral (capsule or oral solution); capsules or oral solution should be swallowed whole with approximately 250 mL (1 glass) of water with or without food but should not be taken with grapefruit or grapefruit juice *Entrectinib* Capsules or oral solution should be swallowed whole with approximately 250 mL (1 glass) of water with or without food but should not be taken with grapefruit or grapefruit juice	CTCAE grade 1 and 2 allowed	≥1 × 10^9^	≥100 × 10^9^/L	CTCAE grade 1 and 2 allowed	Normal liver function	—	No specific prophylaxis Potential interaction with other medications (such as anti-HIV drugs, sartans, statins, antidiabetics, warfarin) to be considered Women using systemically acting hormonal contraceptives should be advised to add a barrier method	Liver function: before the first dose and monthly for the first 3 months of treatment, then periodically during treatment, with more frequent testing in patients who develop transaminase elevations Patients with grade 2 ALT and/or AST increases should be followed with serial laboratory evaluations every 1 to 2 weeks

ALT, alanine transaminase; AST, aspartate aminotransferase; CTCAE, Common Terminology Criteria for Adverse Events; ECG, electrocardiogram; HIV, human immunodeficiency virus; i.v., intravenous; p.o., per os; RT, radiotherapy.

There is relatively little data on the efficacy and risks of vaccination specifically in brain tumor patients. We commonly advise to have vaccinations against influenza and COVID-19, irrespective of whether patients are undergoing treatment or not. To what extent treatments given to brain tumor patients affect the efficacy of the vaccines remains uncertain. There is increasing evidence for a risk of reactivation of viral hepatitis in patient populations at risk.^72^ There is also increased risk of fatal systemic vaccine-associated disease with the use of yellow fever vaccine. Live vaccines are contraindicated in immunosuppressed patients. In case of previous viral disease (hepatitis), a specialized consultation should be planned.

Treatment interactions should be considered, for example, with enzyme-inducing antiepileptic drugs and potentially discussed with pharmacists. Complementary and alternative therapy (including herbal treatments) should be discouraged in the absence of information on potential interactions. Their use should be documented in the medical file of the patient. Further general recommendations for cancer chemotherapy and patient instruction are provided in Notes S2 and S3.

### Steroids

Steroids have been a mainstay in the symptomatic treatment of brain tumor patients for decades. They are mainly used to reduce the tumor-surrounding edema, which is commonly found in different types of brain tumors. By reducing mass effect, steroids provide relief from various symptoms such as headaches, nausea, and many focal neurological deficits. Dexamethasone is most frequently used with daily doses mostly in the range of 2–16 mg.^73^ Despite their undisputed beneficial effects on the patients’ quality of life, steroids are also associated with significant toxicities which ultimately override the positive aspects; furthermore, steroid intake may be associated with inferior survival in glioblastoma.^74^ Treatment with steroids is associated with increased blood sugar levels, particularly in patients with diabetes. These patients should be checked carefully for a hyperglycemic condition that may require appropriate antidiabetic treatment. Similarly, an increase in blood pressure may require regular controls and the initiation or adaption of antihypertensive medication. Insomnia developing during steroid intake may be prevented or reduced by administering steroids as a single dose in the morning, together with sleeping aids, if absolutely needed. Obstructive sleep apnea may develop due to the redistribution of fat. More problematic side effects that typically occur in patients with a continuous need of steroid treatment comprise gastric ulceration, rarely bowel perforation, osteoporosis with the risk of vertebral fractures, myopathy, depression, and less frequently psychosis. Psychiatric disorders related to steroids need to be appropriately treated with antidepressant and neuroleptic drugs and psychiatric consultations may be required. A causative role for comedications such as levetiracetam must be considered. No specific prophylaxis other than reducing the steroid dose is available. In patients at risk for developing osteoporosis, supplementation with calcium and vitamin D formulations is frequently used in the absence of compelling evidence. Myopathy may represent a particular problem for patients with brain tumors as they are frequently affected by neurological deficits such as tumor-related paresis, ataxia, and other symptoms, which may worsen because of myopathy-associated weakness. Physiotherapy may help to prevent falls and maintain quality of life. Because of an increased risk for the occurrence of *Pneumocystis jirovecii* pneumonia, prophylactic treatment with trimethoprim–sulfamethoxazole or pentamidine inhalations should be evaluated in patients requiring steroid treatment for more than 4 weeks or those taking steroids in combination with alkylating chemotherapy or patients low lymphocyte counts.^75^ While the interaction of steroids with immunotherapeutic agents has not yet been fully clarified, there is increasing evidence that concurrent steroid intake interferes with the antitumor activity of immune checkpoint inhibitors.^76^

In order to prevent or revert steroid-induced complications, the lowest possible dose that provides symptom control should be administered for the shortest possible time.

### Temozolomide

Temozolomide is an oral alkylating agent mainly used in the first-line treatment of glioblastoma and in astrocytoma, IDH mutant, and in oligodendroglioma either as part of initial treatment or at relapse.^77^ It is commonly given at 75 mg/m^2^ during radiotherapy 1–2 hours before radiotherapy and in the morning prior to breakfast at 150 mg/m^2^ for the first cycle and 200 mg/m^2^ from the second cycle after radiotherapy (Supplementary Table S4).^78^ The practice to administer temozolomide in the evening rather than in the morning should be discouraged, given that the clinical trial showing efficacy advised administration in the morning. Moreover, preliminary data even indicate inferior activity of temozolomide when given in the evening.^79^

The most common dose-limiting toxicities are hematological, notably thrombocytopenia (Table 2). Bone marrow depression occurring after weeks of daily dosing can last for several weeks, most patients recover and maintenance treatment can still be tried. The temozolomide label recommends routine prophylaxis against *P. jirovecii* pneumonia for all patients during concomitant treatment with RT. However, current clinical practice varies widely regarding this prophylaxis, and routine prophylaxis has been challenged in a population-based report that considered the benefit risk ratio with regard to protection from infection versus induction of myelosuppression by this prophylaxis not favorable.^80^ Based on their analyses, these authors proposed initiating prophylaxis for patients with lymphopenia below 0.25 × 10^9^/L.^81^

**Table 2.** Frequency of CTCAE Grade 3/4 Hematological Toxicity (%) by Drug in Primary Brain Tumor Trials^a^

	Neutropenia	Lymphopenia	Thrombocytopenia	Anemia	References
Temozolomide	7	7	12	1	Stupp et al.^76^
Procarbazine	3	Not specified	4	2	Yung et al.^82^
Lomustine	20	0	25	2.4	Wick et al.^83^
Vincristine	—	—	—	—	No single agent data, probably low risk
PCV	32	Not specified	21	7	van den Bent et al.^84^
Bevacizumab	0	0	0	0	Kreisl et al.^85^
	0	2.3	0	0	Odia et al.^86^
Vemurafenib	—	—	—	2	Larkin et al.^148^
Dabrafinib/tramatenib	10	—	—	—	Wen et al.^87^
Laroctrectinib (*n* = 33, including only 7 adults)	3	Not reported	Not reported	0	Doz et al.^88^
Entrectinib	—	—	—	—	No data

CTCAE, Common Terminology Criteria for Adverse Events.

^a^See dedicated chapters for management of these toxicities and reexposure to therapy.

Nonhematological side effects include nausea, loss of appetite, fatigue, and hepatotoxicity (Table 3). Constipation is also commonly seen in patients on temozolomide but mainly due to the concurrent use of antiemetic agents. Proper bowel management^82^ and reducing the dose or giving antiemetic drugs on alternating days or even less frequently and adaptation of food may alleviate constipation. Hives associated with temozolomide are usually responsive to antihistamine drugs and commonly do not require stopping treatment. Premedication may be considered a few days before starting the temozolomide cycle and a dermatologic consult is advised. In cases of severe allergic reactions desensitization may be considered.

**Table 3.** Frequency of CTCAE Grade 3/4 Nonhematological Toxicity by Drug in Primary Brain Tumor Trials^a^

	Toxicity (%)	%	References	Management and Consequences for Tumor-Specific Treatment
Temozolomide	Fatigue	13	Stupp et al.^76^	Dose reduction
	Skin	3		Pause until resolution, reexpose
	Nausea, vomiting	2		Enforce antiemetic treatment
	Infection	7		Antibiotics
	Hepatotoxicity	Rare		Stop treatment and assess relatedness with hepatologist
Procarbazine	Nausea and vomiting	3	Yung et al.^82^	Dose reduction or stop
	Fatigue	2		Dose reduction
	Rash	25		Stop treatment
Lomustine	Nausea	23	Wick et al.^83^	Enforce antiemetic treatment
	Infection	4		Dose reduction
	Hepatotoxicity	Not reported		Dose reduction or discontinue
Vincristine	Polyneuropathy			Stop in case of signs of polyneuropathy
PCV	Nausea (mostly lomustine related)		Van den Bent et al.^84^ and Wick et al.^149^	Prevent with 5-HT_3_ antagonists
	Rash (grades 2/3)	1–10		Stop procarbazine, regardless of grade
	Polyneuropathy (grades 2/3)	2–7		Stop vincristine once signs appear
	Hepatotoxicity (grades 2/3)	10		Dose reduction of procarbazine first, then lomustine
	Asthenia, lack of appetite, weight loss	5		Mild cases: metoclopramide, if severe: dose reduction or stop procarbazine
Bevacizumab	Hypertension grade 3 or 4	4.2	Gilbert et al.^150^	Treat with antihypertensive medication and continue. Dose reduction recommended depending on severity of antihypertensive medication. Consider extension of treatment intervals up to 4–6 weeks between infusions
		11.3	Chinot et al.^44^	
	Proteinuria	5.4	Chinot et al.^44^	Treatment interruption up to normalization. Consider dose reduction or extension of treatment intervals, eg, 4–6 weeks between infusions
	Fatigue	5–13.1	Gilbert et al.^150^	Consider specific physical activity
	Venous thromboembolic events	4.6	Gilbert et al.^150^	Anticoagulation and continuation of therapy without dose reduction
		7.6	Chinot et al.^44^	
	Arterial thromboembolic events	5	Chinot et al.^44^	Evaluation of stroke etiology, reevaluation of treatment continuation
	Hemorrhage, not further specified	1.6	Gilbert et al.^150^	Treatment discontinuation
	Cerebral hemorrhage grade 4	2	Chinot et al.^44^	Treatment discontinuation
	Bleeding outside the CNS	1.3	Chinot et al.^44^	Treatment interruption. Evaluation of bleeding severity and available clinical strategies. Evaluate dose adaptation or treatment discontinuation
	Wound dehiscence	1.3–1.6	Gilbert et al.^150^	Treatment interruption. Continue without dose reduction after complete wound healing
		3.3	Chinot et al.^44^	
	Nausea and vomiting	1–4.2	Gilbert et al.^150^	5-HT_3_ antagonists, no treatment interruption or dose reduction
	Visceral perforation	1.2	Gilbert et al.^150^	Treatment discontinuation
		1.1	Chinot et al.^44^	
Vemurafenib	Skin	5–15	Larkin et al.^148^	Dose interruption and dose reduction
	Arthralgia	3		
	Liver function	5		
	Fatigue	3		
Larotrectinib (*n* = 33, including 7 adults)	Dysphagia	6	Doz et al.^88^	Dose interruption and dose reduction
	Pneumonia	6		
	Vomiting	3		
	Headache	3		
	Pyrexia	3		
	Urinary tract infection	3		

CNS, central nervous system; CTCAE, Common Terminology Criteria for Adverse Events.

^a^Alternative causes of toxicity should always be explored.

Recommendations for dose modifications are compiled in Supplementary Table S4. Patients with rare hereditary problems of galactose intolerance, total lactase deficiency or glucose–galactose malabsorption should not take temozolomide. Coadministration with valproic acid was associated with a small but statistically significant decrease in the clearance of temozolomide and augments the hematological toxicity of temozolomide.^89^

There are reports of patients who have taken the recommended dose for the regimen for more than 5 days of treatment (up to 64 days) with adverse reactions reported including bone marrow suppression, with or without infection, in some cases severe and prolonged and resulting in death. In the event of an overdose, a hematological consultation is recommended. Supportive measures should be provided as necessary.

#### Procarbazine

Procarbazine with its active metabolite benzyla­zoxyprocarbazine is a brain penetrant oral methylating agent.^84^ In neuro-oncology it is usually given in combination with lomustine (CCNU) and vincristine as part of the procarbazine CCNU vincristine (PCV) regimen. The most relevant toxicities are hematological and gastrointestinal, mainly nausea and an increasing loss of appetite leading to weight loss; severe vomiting is infrequent.^90^ A hypersensitivity rash is a frequent side effect of procarbazine (up to 25% of patients) which typically does not respond to steroids and antihistaminic agents. Because procarbazine exhibits monoaminoxidase inhibitory activity, the package inserts warns against food with high tyramine content food (wine, yoghurt, cheese, bananas). The use of procarbazine together with tricyclic antidepressants may rarely result in a serotonin syndrome that requires recognition and cessation of either agent.^91^ When given within the “classical” PCV schedule, procarbazine is given on days 8–21 at a dose of 60 mg/m^2^ orally.^92^ Procarbazine dose should be dose reduced in steps of 25% in case of hematological toxicity and in case of significant lack of appetite, asthenia, and weight loss. In case of rash its use should be discontinued (Tables 2 and 3).

#### Nitrosoureas

Nitrosoureas are a class of alkylating agents frequently used for glioma. They are highly lipid soluble and thus brain penetrant. The most well-known nitrosoureas are lomustine (CCNU), carmustine (BCNU, for i.v. use), fotemustine and nimustine (ACNU). Lomustine is the most frequently used nitrosourea.^93^ It is part of the PCV regimen and frequently used as a single agent in recurrent glioblastoma. Lomustine is contraindicated in case of celiac disease or wheat allergy. Patients with hereditary galactose intolerance, total lactase deficiency, or glucose galactose malabsorption should not take lomustine. A delayed and cumulative myelosuppression is the main side effect of this class of agents affecting platelets more than white blood cell count.^94^ An interstitial pneumonitis has been described after carmustine use, but is exceedingly rare with lomustine.^94,95^ Despite that, monitoring pulmonary function is part of the package insert. However, the absence of relevant pulmonary toxicity in lomustine-treated patients in clinical practice and in a review of toxicity data from clinical trials^93,96^ does not support routine lung function monitoring in asymptomatic patients without a history of pulmonary disease. Nausea is common but mild and usually easily controlled with antiemetics. Mild and transient serum aminotransferase elevations are frequent. Lomustine is metabolized by the hepatic cytochrome P450 system, patients on enzyme-inducing antiepileptic drugs or other enzyme-inducing agents should therefore be switched to other drugs. Severe renal insufficiency is also a contraindication to lomustine.

When given as a single agent the dose is usually 110 or 130 mg/m^2^ in cycles of 6 weeks because of the delayed myelosuppression (Supplementary Table S5). Most centers cap the dose at 200 mg. In case of significant hematological toxicity experienced during temozolomide treatment in the first-line setting, a first lomustine cycle with a reduced dose reduction can be considered and escalation at cycle 2 if the first cycle was well tolerated.

### Vincristine

Vincristine belongs to the vinca alkaloid compounds and is incorporated in several polychemotherapy oncology protocols. Its most prevalent use for the treatment of primary brain tumors is in combination with procarbazine and lomustine in the PCV regimen.^97,98^ Vincristine has a very poor oral bioavailability and is thus administered intravenously. It shows poor blood brain barrier penetration and might thus only reach contrast-enhancing tumor areas in the CNS (with a leaky blood–brain barrier). The main metabolism occurs in the liver via the cytochrome enzyme system, particularly CYP3A4 and CYP3A5. Half time is around 80–85 hours, yet, interpatient variability in metabolism as well as drug–drug interactions need to be considered.^99^

Vincristine alone is rarely myelosuppressive. Of note, its microtubulin target is also a critical component of nerve fiber axons. Thus, the main treatment-induced clinical complications are axonopathies leading to axonal sensorimotor neuropathies of large and small nerve fibers with symptoms like numbness, tingling, neuropathic pain, and hyporeflexia. Autonomic neuropathy may manifest with constipation. Risk factors for the development of vincristine-induced neuropathies include preexisting hereditary neuropathies, and thus a corresponding family history should be considered as a contraindication for vincristine treatment. Even though preexisting vitamin deficiencies are associated with neurotoxicity, neither monitoring nor preventive vitamin supplementations are appropriate.^100^ Since there are no standardized recommendations how to treat vincristine-induced polyneuropathy,^87^ early recognition and cessation of vincristine exposure are essential. Medications with duloxetine, venlafaxine, or baclofen might be helpful in patients with painful neuropathy.

#### Bevacizumab

Bevacizumab is a recombinant humanized IgG1 monoclonal antibody targeting vascular endothelial growth factor (VEGF)-A. Its mode of actions includes reduced vessel leakiness and intratumoral pressure, as well as vessel regression. The reduction of vasogenic edema results in corticosteroid-sparing effects. The standard dose for glioblastoma per label is 10 mg/kg, but de-escalation to 7.5 or 5 mg/kg and prolonged dosing intervals can be considered on an individual basis. The half time is in the range of 18–21 days, thus infusions are given every 14–21 days depending on the regimen. Bevacizumab prolongs progression-free survival but not overall survival in newly diagnosed and progressive glioblastoma and has been approved for recurrent glioblastoma in the United States, Switzerland, and Japan, although not by the European Medicines Agency.

The most common side effects with bevacizumab are hypertension, hoarseness, fatigue or asthenia, diarrhea and abdominal pain, and proteinuria. The most serious side effects are gastrointestinal perforation, hemorrhage, impaired would healing and arterial thromboembolism.

The management of bevacizumab-related adverse events included prolongation of infusion intervals (up to 8 weeks) and/or discontinuation (if the interval exceeded 8 weeks). Further relevant aspects for clinical management are wound healing, thus a latency of 28 days (i.e. exceeding one half time of the drug) before/after major surgical interventions should be considered. No guidelines are available regarding bevacizumab in case of elective minor surgery (dental surgery, central venous access). A wound dehiscence was noted in 6 of 195 patients (3%) receiving bevacizumab within 10 days of port placement.^101^ Treatment adjustments after adverse events are recapitulated in Table 3.

### Targeted Therapy

#### BRAF

A BRAF V600E mutation is present in 3%–4% of gliomas, for which treatment with BRAF inhibitors (eg, vemurafenib) alone or combined with MEK inhibition, for example, trametinib with dabrafenib, may be effective.^102,103^ The addition of an MEK inhibitor to BRAF inhibition improves the blockade of the mitogen-activated protein kinase pathway which mitigates acquired resistance against BRAF monotherapy and reduces the incidence of skin-related toxicities of BRAF inhibition, in particular verrucal keratosis.^104^ Vemurafenib is associated with arthralgias and skin toxicity in more than half of the patients, and with photosensitivity in 40%. It is given at a dose of 960 mg twice per day continuously in 28-day cycles, with dose reductions on the basis of toxicity in decrements of 240 mg at each dose administration.^102^ The skin toxicity requires avoidance of sun exposure and use of sunblocks. Combination treatment dabrafenib (150 mg twice daily) and trametinib (2 mg once daily orally) is associated with mild fatigue (40%), nausea and vomiting (30%), pyrexia (30%) and rash (30%); nasophayngitis, constipation, and arthralgias are also relatively frequent but mild.^103^ Neutropenia grade 3 may occur in 10%. The combination is given at a starting dose of 150 mg orally twice daily and 2 mg orally once daily, respectively, with dose reductions and delays in case of toxicities. Cardiac monitoring is required (see below, Table 1). Given the frequent requirements for the management of pyrexia and the rarity of BRAF mutations, treatment should be done in collaboration with a specialist with experience in these agents.^105,106^

#### NTRK

Oncogenic fusions of the neurotrophic tyrosine receptor kinase (*NTRK*) genes NTRK1, NTRK2, and NTRK3 are observed with various gene partners and oncogenic drivers.^107^ Among adults with primary brain tumors, NTRK fusions are reported with a frequency of 1%–2%^108,109^ and are most frequently found in glioblastoma.

Larotrectinib and entrectinib represent the first generation of NTRK inhibitors and are approved by FDA and EMA in TRK fusion-positive cancers. Fatigue, liver enzyme increases, cough, constipation, diarrhea, dysgeusia, dizziness, nausea, and vomiting have been reported in more than 20% of patients in the larotrectinib trials.^110,111^ The most frequent adverse events reported in the entrectinib trials include weight gain, arthralgia and myalgia, dysgeusia, dysesthesia and peripheral sensitive neuropathies, dizziness and ataxia, fatigue, constipation, diarrhea, peripheral edema nausea, and vomiting.^111,112^ Risks of congestive heart failure, QTc prolongation, fractures have to be considered with entrectinib. Proposed assessment before treatment initiation and during follow-up are described in Table 1. Second generation drugs such as selitrectinib and repotrectinib are currently evaluated in clinical trials. Adverse reactions may require temporary interruption, dose reduction, or discontinuation. Concomitant use of strong or moderate CYP3A inhibitors increases entrectinib plasma concentrations whereas strong or moderate CYP3A inducers may decrease entrectinib plasma concentrations. Concomitant medication should be regularly monitored.

### Main Expert Recommendations

For young adults, both females and males, specialized consultation should be offered to discuss fertility and potential solutions prior to initiation of systemic pharmacotherapy.^113^
The relevance of an adequate contraception should be discussed with both male and nonmenopausal female patients before treatment initiation. Pregnancy tests should performed in case of any doubt on a potential pregnancy before and during the conduct of the systemic pharmacotherapy.^113^
Systemic pharmacotherapy should commonly not be given unless neutrophil counts are >1500/µL and platelet counts >100 000/µL.
Pharmacological interactions, notably with antiepileptic drugs, should be considered when initiating pharmacotherapy for any brain tumor.
Complementary and alternative therapy (including herbal treatments) should be discouraged, especially during systemic pharmacotherapy, and always documented in the patient charts.
Systemic pharmacotherapy should commonly not be given when aspartate aminotransferase and alanine aminotransferase levels are elevated threefold or more.
Pulmonary function should be checked prior to nitrosourea therapy in patients with chronic pulmonary disease.
Vincristine should be stopped upon the first symptoms or signs of polyneuropathy and reexposure is not advised.
Cardiac monitoring, including electrocardiogram (ECG) and left ventricular ejection fraction evaluation, is required before and during treatment with BRAF and NTRK inhibitors.
Comedication should be taken into account notably for NTRK inhibitors.

## Cancer Pharmacotherapy—Management of Side Effects by Organ Site

### Hematological Toxicity

#### Neutropenia

The decrease of the neutrophil count below normal is the most common side effect of the chemotherapeutic agents used in primary brain tumors (Supplementary Table S6). The agents used in gliomas, temozolomide, lomustine, and less so procarbazine frequently cause neutropenia, as can etoposide, carboplatin, and cyclophosphamide which are used in other primary brain tumors. Grading of neutropenia is based on the CTCAE scale^1^ and dose adjustments for subsequent cycles are performed according to the grading of toxicity. Of note, subsequent myelotoxic chemotherapy regimens may need to be initiated at reduced dose if significant myelotoxicity has occurred during prior treatments.

Absolute neutrophil counts below 500/µL increase the risk of infection and thus febrile neutropenia, hospitalization, and mortality, and this risk increases with longer duration of neutropenia. Since therapy reintroduction is recommended only after the absolute neutrophil count has recovered to >1500/µL depending on the patient and chemotherapy agent, prolonged neutropenia causes dose delays and dose reductions, possibly affecting efficacy.

For these reasons, the use of recombinant granulocyte colony-stimulating factors (G-CSF) is indicated when the risk of febrile neutropenia is higher than 20% or when significant dose reductions and delays occur, or for febrile neutropenia risk of >10% in patients >65 years of age or patients with serious comorbidities. Risk calculation can be done using special models such as the Multinational Association for Supportive Care in Cancer (MASCC) model^114^ or by the use of the National Comprehensive Cancer Network (NCCN) guidelines.^115^

The risk of febrile neutropenia increases with age and is influenced by comorbidities of the patient, the chemotherapeutic agents used, the number of previous lines of therapy, and the fields of prior radiotherapy.^116^ Depending upon the indication for chemotherapy, curative versus palliative, G-CSF prophylaxis may be considered to maintain dose intensity, but this rarely applies to patients with primary brain tumors.

#### Lymphopenia

Lymphopenia is a relatively frequent complication of temozolomide, and less so of other chemotherapy agents used in neuro-oncology. A very low CD4 count (<200/µL), considered absolute lymphopenia, may be encountered, thus predisposing patients to opportunistic infections such as *P. jirovecii* pneumonia or viral infections such as cytomegalovirus.^117^ These risks are aggravated by the chronic use of steroids and the use of radiotherapy. Thus patients with primary brain tumors are prone to these infections and many centers use prophylactic trimethoprim (80–160 mg orally daily or 160 mg 3 times/week on alternate days)/sulfamethoxazole (800 mg) during radiotherapy (even if the absence of concomitant steroids).^118^ Alternatively, regular testing of CD4 counts may permit the use of this prophylaxis only when counts drop below 200/µL.^118^ The discontinuation of prophylaxis is dependent both on the counts and on other parameters such as the use and tapering of steroids. Clinician awareness of these complications is of paramount importance so that diagnosis of pneumocystis pneumonia is made and therapy is initiated promptly.

In patients with an allergy to trimethoprim/sulfamethoxazole or glucose-6-phosphate dehydrogenase deficiency (G6PD) deficiency, close observation can be used although an alternative is inhaled pentamidine or atovaquone.^119^

Although some centers consider antiviral prophylaxis, there is no evidence for its activity so we do not recommend this. No measures can ensure that CD4 counts will rise, nor is dose adjustment advised for this complication. Since higher dose and dose-intense regimens of temozolomide cause more lymphopenia, their use is discouraged, as should be the use of corticosteroids without clear rationale.

#### Thrombocytopenia

During concomitant radiotherapy and temozolomide in patients newly diagnosed with a glioblastoma thrombocytopenia of any CTCAE grade is observed in more than 25% and grade 3/4 thrombocytopenia in 5% which may last for several weeks. During maintenance temozolomide, thrombocytopenia of any grade is reported in 45% and grade 3/4 in 7%.^120^ In the first recurrence setting, thrombocytopenia of any grade was noted in 26% of patients treated with lomustine alone and in 33% of patients treated with both lomustine and bevacizumab.^121^ In the EORTC 26951 trial, grade 3 or 4 thrombocytopenia was noted in 21% of patients treated with PCV after radiotherapy.^92^

Thrombocytopenia is associated with a risk of cerebral and extra-CNS hemorrhage, chemotherapy dose reductions, dose delays, or discontinuation. Most institutional recommendations and clinical trial protocols state that a platelet count of 100 × 10^3^/µL is required for the continuation of chemotherapy and that CTCAE grade 3 or 4 toxicities should trigger a reduction of chemotherapy to the next lower dose level. Prophylactic platelet transfusions should be administered if platelet counts are below 10–20 × 10^3^/µL, or between 20 × 10^3^/µL and 50 × 10^3^/µL in the presence of bleeding events, such as exteriorizing bleeding, hematoma, or petechiae, as per ASCO guidelines.^122^ Simple recommendations such as avoiding the use of sharp object (steak knife), avoiding sports at risk, avoiding food associated with a risk of obstipation, using soft toothbrush or mouthwash, and using an adapted toothpaste should be provided. In case of drug reexposure after dose/schedule modification or discontinuation for thrombocytopenia, a weekly platelet count should be performed. The thrombopoietin receptor agonist, romiplostim, may allow to complete a course of alkylating chemotherapy if thrombocytopenia is the main limiting toxicity,^123^ however, further data are needed.

#### Anemia

Anemia may be caused by the use of chemotherapy, including the agents used in primary CNS tumors, mainly temozolomide, procarbazine, and lomustine, as well as the platinum agents and cyclophosphamide used in ependymoma and medulloblastoma. In cases where anemia is sudden or unexplained, a work-up to rule out other causes of anemia such as blood loss, hemolysis, or renal failure should be performed.

If hemoglobin levels drop below 8 g/dL, blood transfusion is indicated.^124^ This level of hemoglobin may be different in patients with comorbidities such as pulmonary, cerebrovascular, or cardiac disease or in patients who are symptomatic with severe fatigue, arrhythmia, chest pain, or dyspnea.

Most primary brain tumors that are treated with chemotherapy are not treated with curative intent. In such cases the use of erythropoiesis-inducing agents, where available, may be used to avoid transfusions. Of note, these agents are used to prevent anemia more than to treat it, as it may take weeks for an erythropoiesis-inducing agent to correct anemia while a transfusion will correct it immediately. The usual cutoff for introducing such agents is a hemoglobin of 10 g/dL, but lower threshold levels may be used to reduce cost and the risk of side effects. The goal should be a hemoglobin level that does not require transfusion and once this goal is achieved or a rise of 1 g/dL of hemoglobin in 2 weeks is detected, the dose of the erythropoiesis-inducing agent can be reduced by 40% in the case of darbepoetin and 25% for epoetin alpha, as the level of hemoglobin should not surpass 12 g/dL.

Concerns that signaling via the erythropoietin (EPO) receptor expressed on tumor cells may promote tumor progression have not been substantiated.^125^ The use of erythropoiesis-inducing agents may be appropriate to improve quality of life since tumor-promoting claims have not been substantiated.^126^ Anemia is usually not a reason to decrease the dose of, or to withhold, chemotherapy, but dose reduction should be considered usually if transfusions are required repeatedly.

Of note, erythropoietin use may increase the risk of thrombosis as can transfusions, a side effect of importance in immobilized brain tumor patients.^127^ Lastly erythropoiesis-inducing agents may cause arterial hypertension,^128^ a particularly relevant side effect in patients on steroids or bevacizumab.

### Nonhematological Toxicity

#### Skin

Cutaneous adverse drug reactions are infrequent in patients with primary brain tumors receiving antitumor therapy. Skin toxicity is rare with temozolomide whereas procarbazine may cause allergy and occasionally toxic epidermal necrosis^88,129^ which requires cessation of the drug. Furthermore, skin irritations are common but mostly mild in patients who are using TTFields.^130^ Targeted therapies can cause skin reactions ranging from mild skin rash to life-threatening severe cutaneous adverse reactions such as Stevens–Johnson syndrome, toxic epidermal necrosis and the hand-foot skin reaction (HFSR). This toxicity may reflect direct skin toxicity or drug hypersensitivity reactions. Patients treated with BRAF inhibitors may experience photosensitivity and maculopapular eruptions. The same type of cutaneous adverse drug reactions is commonly seen in patients treated with NTRK inhibitors such as larotrectinib^131^ or entrectinib,^112^ particularly cutaneous rash and photosensitivity reactions. Measures for the prevention of HFSR include the removal of preexisting hyperkeratotic areas or calluses and the use of shoe cushions and gloves to prevent pressure-induced stress to soles and palms. Once diagnosed, the management of HFSR includes the use of keratolytic and moisturizing creams for symptomatic relief. In case of severe or persistent symptoms, dose reductions as well as temporary or permanent treatment interruption must be considered.

#### Lung

Pulmonary toxicity is a known complication associated with the use of nitrosoureas. Treatment with carmustine (BCNU) bears a risk for the development of pulmonary fibrosis which occurs within weeks to months after treatment in up to 30% of patients. In rare cases in adult patients with brain tumors, late manifestations of lung fibrosis have been reported.^132^ Younger age and higher nitrosourea doses are associated with an increased risk for pulmonary fibrosis. For carmustine, doses beyond 1500 mg/m^2^ in adults should be avoided. The incidence of pulmonary toxicity in glioma trials is overall very low^133^ perhaps because of the routine use of lomustine which harbors a lower risk for pulmonary toxicity than carmustine.^96^

Infections, such as bronchitis, lower respiratory tract infection or pneumonia are diagnosed in approximately 10%–15% of adult and pediatric patients treated with NTRK inhibitors. Patients who are treated with dabrafenib in combination with trametinib have a risk for the development of interstitial lung disease or pneumonitis.^134^ Currently, no dose modification of dabrafenib is recommended when taken in combination with trametinib for cases of interstitial lung disease or pneumonitis, but treatment should be halted until full recovery from clinical symptoms and reexposure is not advised.^135^ Furthermore, cases of sarcoidosis have been reported in patients receiving dabrafenib and trametinib, mostly involving the skin, lung, eye, and lymph nodes.

#### Liver

Hepatic toxicity has been reported for temozolomide and may include increased liver enzymes, hyperbilirubinaemia, and cholestasis.^88^ Monitoring liver function before and during temozolomide treatment, for example, every 4–8 weeks, is recommended. Abnormal liver function tests should trigger the exclusion of changes in coagulation parameters. Furthermore, HCV infection should be ruled out by virus serology and abdominal sonography should be obtained to confirm that TMZ is the main cause of liver dysfunction. Laboratory abnormalities should be managed with dose reductions, treatment interruption, or discontinuation. Meulengracht’s disease is no contraindication for chemotherapy. Advice from hepatology experts should be sought before potentially active antitumor treatment is halted or discontinued.

Elevated alanine aminotransferase (ALT) and aspartate aminotransferase (AST) values are common findings patients receiving NTRK inhibitors, mostly occurring in the first 3 months of treatment. Interruption or discontinuation of these drugs should be considered depending on the severity of the liver injury. For BRAF inhibitors, liver toxicity, sometimes severe, has been reported.^134^ For antiangiogenic drugs, especially regorafenib, liver function abnormalities have been frequently observed. Therefore, it is recommended to perform liver function tests before initiation of treatment and to monitor these parameters closely. Mild, indirect (unconjugated) hyperbilirubinemia may occur in patients with Gilbert’s syndrome and is no contraindication for chemotherapy. Similar to other drugs which exert liver toxicity, dose modification as well as treatment interruption or discontinuation have to be considered.

#### Kidney

Among classical chemotherapeutic agents used in brain tumor patients, platinum-based drugs have a well-known risk to exert renal toxicity. Nephrotoxicity is more frequently associated with the use of cisplatin compared with carboplatin and elderly patients are more likely to suffer from renal toxicity.^136^ Treatment with bevacizumab may result in renal thrombotic microangiopathy which may manifest as proteinuria.^137^ The severity of the latter may range from clinically asymptomatic, transient, trace proteinuria to nephrotic syndrome. Grade 3 proteinuria was reported in up to 10.9% of treated patients. Grade 4 proteinuria (nephrotic syndrome) was observed in 1.4% of treated patients. Testing for proteinuria is recommended prior to the initiation and before each administration of bevacizumab therapy. This can be done by screening using a dipstick first and in case of 2+ a 24-hour urine analysis. In clinical trials, urine protein levels of ≥2–3 g/24 hours led to the holding of bevacizumab until recovery to < 2 g/24 hours. Proteinuria has been reported rarely in patients treated with regorafenib. Cases of renal toxicity have also been reported upon treatment with vemurafenib ranging from creatinine elevations to acute interstitial nephritis and acute tubular necrosis. Serum creatinine elevations were mostly mild (>1–1.5× ULN) to moderate (>1.5–3× ULN) and usually reversible.

#### Cardiovascular

Dose-dependent arterial hypertension has been observed in bevacizumab-treated patients.^138,139^ Preexisting hypertension should be adequately controlled before starting bevacizumab treatment and monitoring of blood pressure values is recommended during therapy. Bevacizumab should be permanently discontinued if significant arterial hypertension cannot be adequately controlled with antihypertensive therapy, or if the patient develops hypertensive crisis or hypertensive encephalopathy.

Syncope was reported in 5% of patients treated with NTRK inhibitors, sometimes associated with arterial hypotension, dehydration, or QTc prolongation. Similarly, QTc prolongation may also occur in patients treated with BRAF inhibitors.^134^ QTc prolongation may lead to an increased risk of ventricular arrhythmias including *Torsade de Pointes*. Therefore, treatment with these drugs is not recommended in patients with uncorrectable electrolyte abnormalities, including magnesium, long QT syndrome or in patients taking other drugs which may prolong the QTc interval. ECG and electrolytes must be monitored in all patients before and during treatment with BRAF inhibitors. Moreover, if QTc exceeds 500 ms, BRAF inhibitor treatment should be temporarily interrupted. Regorafenib has been associated with an increased incidence of myocardial ischemia and infarction. Therefore, patients with a history of ischemic heart disease should be monitored for clinical signs and symptoms of myocardial ischemia.

#### Gastrointestinal

Gastrointestinal side effects are frequent in patients receiving treatment with classical alkylating agents such as temozolomide, procarbazine, or nitrosoureas. Nausea and vomiting have been reported in 50% of patients or more, depending on the dose and schedule.^140^ Appropriate prophylaxis with antiemetics such as 5HT_3_-receptor antagonists or other drugs will prevent nausea and vomiting in the majority of patients.^141^ In turn, 5HT_3_-receptor antagonists harbor a risk for the development of constipation, which may require appropriate dietary changes or treatment with laxatives. Of note, 5HT_3_-receptor antagonists can prolong the QTc interval.

Patients treated with bevacizumab have an increased risk of gastrointestinal perforation. In such cases, therapy should be permanently discontinued. Most targeted agents frequently exert gastrointestinal side effects such as diarrhea, mostly of mild-to-moderate intensity. Diarrhea, nausea, and vomiting are commonly seen during therapy with BRAF and MEK inhibitors and can be accompanied by abdominal pain and gastrointenstinal bleeding.^134^

#### CNS

Neurocognitive impairment is frequently diagnosed in brain tumor patients.^142^ Beyond direct damage caused by the tumor and sequelae of surgery and radiotherapy, many chemotherapy as well as targeted anticancer drugs may contribute to undesired effects, also referred to as “chemobrain”, but this syndrome plays almost no role in patients with primary brain tumors. Currently, no specific measures are available to prevent this condition. There have been reports of bevacizumab^143^ and regorafenib^144^ treated patients developing posterior reversible encephalopathy syndrome (PRES), a rare neurologic disorder which may present with seizures, headache, altered mental status, visual disturbance, or cortical blindness, with or without associated hypertension. PRES is diagnosed based on typical clinical and MRI findings. Other reasons for the observed clinical symptoms and signs need to be ruled out. In patients developing PRES, symptomatic treatment of seizures and antihypertensive therapy as needed are recommended along with discontinuation of the causative agent. The safety of reinitiating antiangiogenic therapy in patients previously experiencing PRES is not known.

#### Fertility

Chemotherapy, particularly alkylating agents, can cause infertility in men and women, the older the patient the higher the risk of infertility, particularly for women over 40 years old. This issue is especially important for young patients with favorable prognosis or potentially curable brain tumors. As chemotherapy-induced infertility cannot be prevented, measures should be taken for fertility preservation. A few studies have shown benefit from the use of luteinizing hormone releasing hormone analogs during chemotherapy in young women thus inducing a state of dormancy in the ovaries, preventing gonad toxicity from chemotherapy.^145^ Furthermore, sperm banking for men and oocyte, embryo or ovarian tissue cryopreservation may be an option for patients before the start of chemotherapy,^146^ for example, in medulloblastoma during the radiotherapy phase or prior to treatment in patients with oligodendroglioma and astrocytoma, IDH mutant. Embryo cryopreservation is a well-known technique, oocyte cryopreservation is more recent and unstimulated ovarian tissue cryopreservation has started being used.^83,147^ The collection of oocytes usually requires hormonal stimulation. In most countries a legal framework addressing ownership issues controls the ex vivo fertilization of oocytes.^85^ Infertility can also be a result of hypophyseal insufficiency caused by radiotherapy and in very rare cases by inadvertent radiation to the ovaries during neuraxis radiotherapy. For the former endocrine evaluation is of paramount importance. For the latter, in the rare occasions where the patient’s body habitus is such that the field may involve the ovaries, the solution is oophoropexy which is the transposition of the ovaries before RT to protect them from the radiotherapy.^86^ Therefore radiotherapists need to assess this while designing the field and act accordingly.

### Main Expert Recommendations

Absolute neutrophil counts below 500/µL should trigger antibiotic prophylaxis and consideration of treatment with G-CSF.
A CD4 T-cell count below 200/µL should trigger prophylaxis with trimethoprin sulfamethoxazole, and acyclovir.
Thrombocytopenia below 10–20 × 10^3^/µL should be treated with platelet transfusions.
Red blood cell transfusions should be considered when hemoglobin levels fall below 8 g/µL.
Patients with a history of cardiopulmonary disease should undergo lung functional testing prior to treatment with nitrosourea.
Dose reductions or discontinuation of cancer pharmacotherapy need to be considered for patients with severe or recurrent hepatotoxicity.
Counseling for fertility should be offered for most women and men prior to the start of cancer pharmacotherapy.

## Conclusions

The quality of patient management and care depends to a relevant degree on how adverse events of therapy are recognized, managed, and prevented. Neuro-oncologists should consider working closer with palliative care specialists and symptom management services to be proactive in addressing side effects of cancer therapy. The present recommendations represent a contemporary consensus. Patterns of managing and responding to toxicity with changes in the treatment regimen may vary significantly across the globe. However, we believe that this article may represent a first important step to harmonize this hitherto somewhat neglected field of neuro-oncology.

## Supplementary Material

noad038_suppl_Supplementary_Material
