# Supplementary material for: Diagnosis and management of complications from the treatment of primary central nervous system tumors in adults
Source: Neuro Oncol. 2023 Feb 27;25(7):1200–24. doi: 10.1093/neuonc/noad038 (PMC10326495; doi:10.1093/neuonc/noad038)
Supplement: noad038_suppl_Supplementary_Material [file noad038_suppl_supplementary_material.docx]

**Diagnosis and management of complications from the treatment of primary central nervous system tumors**

**Supplementary Material**

Note S1. General recommendations for cancer pharmacotherapy

Note S2. Additional general recommendations for cancer pharmacotherapy

Note S3. Monitoring of pharmacotherapy: instructions for patients and caregivers

Table S1. The Landriel Ibañez Classification for neurosurgical complications including examples relevant to neurosurgical oncology

Table S2. Management of antithrombotic and anticoagulant therapy in patients receiving these treatments prior to surgery for a brain tumor

Table S3. Treatment of TTFields-associated dermatologic adverse events.

Table S4. Recommendations for dose modifications for temozolomide

Table S5. Recommendations for dose modifications for lomustine

Table S6. Management of hematological toxicity

Note S1. General recommendations for cancer pharmacotherapy.

In general, capsules must not be opened. If a capsule is damaged, the powder it contains must not come into contact with the skin or mucous membranes, or be inhaled or ingested. In case of skin contact, the affected area should be washed with water; in case of contact with eyes, they should be rinsed with water. Unused medicine or waste should be disposed of according to institutional and drug-specific guidelines. Specifically, pregnant women should not be in contact with cancer pharmacotherapy. Drugs should be access-restricted at home to protect children and animals. Oral agents should be stored in the original bottle in order to protect them from moisture and should not be stored at more than 25°C. Appropriate steps should be taken to avoid exposure. This includes appropriate equipment, such as wearing gloves, and washing hands with soap and water after handling of such products. Standard operating procedures to manage extravasation must be available in the unit where infusions are administered.

Note S2. Additional general recommendations for cancer pharmacotherapy.

The treatment related embryofetal risk should be explained to the patients. Women of childbearing potential are defined as premenopausal females capable of becoming pregnant. A highly effective method of birth control (low failure rate less than 1% per year when used consistently and correctly) must be discussed with the patients. These methods include for women: combined (estrogen and progestogen containing) hormonal contraception associated with inhibition of ovulation (oral, intravaginal, transdermal); progestogen-only hormonal contraception associated with inhibition of ovulation (oral, injectable, implantable); intrauterine device; intrauterine hormone-releasing system; bilateral tubal occlusion; vasectomized partner; sexual abstinence and for men: vasectomy; sexual abstinence; condoms during treatment and for at least 6 months after the last dose. A female partner of childbearing potential should use highly effective birth control methods.

The medical history should be checked prior to treatment initiation and adverse events but also any intercurrent events (such as infection, venous thrombo-embolic events) that could contra-indicate the administration of the treatment should be checked prior each treatment administration during the conduct of the treatment. The weight of the patients should be checked prior to each drug administration if the dose of the agent depends on the weight of the patients. Modification of more than 10% should be reflected in the prescribed dose. The agent related blood testing should be obtained within 24-48 hours prior to drug administration. In case of a clinical doubt on progression, an imaging assessment and adequate testing should be performed, ideally before drug administration.

Note S3. Monitoring of pharmacotherapy: instructions for patients and caregivers

The secure administration of oral treatments in patients with brain tumors can be challenging, given potential cognitive and neurological physical impairment. A patient diary may be of help, but does not exist in standardized format. Help from caregivers may be required for documentation of outpatient treatment. Missed doses of oral treatment should be noted in the patient diary, but should not be replaced. Patients who vomit may be redosed only if the capsules are still intact and visible in the vomitus and after appropriate antiemetic therapy has begun. Oral treatment should be taken approximately at the same time on each dosing day. In case of inadvertent ovredoseing, the treating physician should be informed without delay.

Table S1. The Landriel Ibañez Classification for neurosurgical complications including examples relevant to neurosurgical oncology (Adapted from Landriel Ibañez et al., 2011 ^1^).

| **Grade** | **Definition** | **Examples for neurosurgical oncology** |
| --- | --- | --- |
|  |  |  |
| Grade I | Any non–life-threatening deviation from normal postoperative course, not requiring invasive treatment |  |
|  |  |  |
| Ia | Complication requiring no drug treatment | - Transient new neurological deficit - Subgaleal CSF collection |
|  |  |  |
| Ib | Complication requiring drug treatment | - Seizures requiring anticonvulsants - CSF infection requiring antibiotics |
|  |  |  |
| Grade II | Complication requiring invasive treatment such as surgical, endoscopic, or endovascular interventions |  |
|  |  |  |
| IIa | Complication requiring intervention without general anaesthesia | - CSF fistula requiring lumbar puncture - Subgaleal CSF collection requiring lumbar drainage |
|  |  |  |
| IIb | Complication requiring intervention with general anaesthesia | - CSF leaks requiring surgical repair - Subgaleal CSF collection requiring shunt drainage - Wound and bone flap infection requiring debridement and removal |
|  |  |  |
| Grade III | Life-threatening complications requiring management in Intensive Care Unit (ICU) |  |
|  |  |  |
| IIIa | Complication involving single organ failure | - Acute hydrocephalus requiring external ventricular drainage - Intracerebral haematoma requiring re-operation |
|  |  |  |
| IIIb | Complication involving multiple organ failure | - Meningitis and pneumonia - Intracranial hypertension and haemodynamic instability |
|  |  |  |
| Grade IV | Complication resulting in death | - Death |
|  |  | |
| Surgical complication | Adverse events that are directly related to surgery or surgical technique | |
|  |  | |
| Suffix “T” (transient) | New neurologic deficit improving within 30 days of surgical procedure; can be added to each grade of complication | |
|  |  | |
| Suffix “P” (permanent) | New neurologic deficit extending beyond 30 days of surgical procedure; can be added to each grade of complication | |

Table S2. Adaptation of anti-platelet agents and anticoagulant therapy in patients scheduled for brain tumor surgery (expert opinion)

| **Agent** | **Interruption before brain tumor resection or biopsy** | **Vascular risk management during the interruption** | **Re-introduction after brain tumor resection or biopsy** |
| --- | --- | --- | --- |
|  |  |  |  |
| Anti-platelet agents | 5 days | prophylactic dose LMWH | 2 weeks |
|  |  |  |  |
| **Anticoagulants** |  |  |  |
| Heparin or LMWH (prophylactic dose) | none | continue | continue dependent on indication for heparin |
| Heparin or LMWH (therapeutic dose) | 1 day | prophylactic dose LMWH | dependent on indication for heparin |
| Vitamin K antagonists | 1-2 weeks | prophylactic dose LMWH | 2 weeks |
| DOAC | 2 days | prophylactic dose LMWH | 2 weeks |

Abbreviation: DOAC: direct oral anticoagulant; LMWH: low molecular weighted heparin

Table S3. Treatment of TTFields-associated dermatologic adverse events (expert opinion).

| **Type of dermatologic**  **Adverse event** | **Management of grade 1 adverse event** | **Management of grade 2 adverse event** | **Management of grade 3 adverse event** |
| --- | --- | --- | --- |
|  |  |  |  |
| Dermatitis | Topical corticosteroids | Avoid direct contact of arrays with affected skin, high-dose topical corticosteroids | Treatment interruption |
|  |  |  |  |
| Erosions | Topical antibiotics (e.g. mupirocin) | Avoid contact of arrays and adhesive tape with affected skin  Topical and oral antibiotics | Treatment interruption, review concomitant medication; dermatology consultation |
|  |  |  |  |
| Infections | Culture and corresponding  topical and/or oral antibiotics | Avoid contact of arrays and adhesive tape with affected skin.  Culture and treat with corresponding antibiotics (oral or topical) | Dermatology consultation  Review concomitant medication  Skin culture and oral antibiotics;  Consider treatment interruption |
|  |  |  |  |
| Ulceration | Culture of open areas and corresponding  topical antibiotics | Avoid contact of arrays and adhesive tape with affected skin.  Culture and treat with corresponding antibiotics (oral or topical) | Treatment interruption  Dermatology consultation |

Adapted from Murphy J et al., 2016 ^2^

* Five subtypes of dermatologic adverse events have been described: hyperhidrosis, xerosis or pruritus, contact dermatitis, skin erosions and ulcers, skin and soft tissue infections ^3,4^. Risk factors included (1) prior craniotomies, particularly with transducer array placement over surgical scar lines or surgical hardware from craniotomy repair; (2) patients with excessive sweating; (3) patients with persistent alopecia and (4) skin exposure to ultraviolet and/or ionizing radiation. Combination with temozolomide or bevacizumab or high doses of corticosteroids or oral antibiotics such as penicillin or cephalosporin might increase the risk of dermatologic adverse events. This is due to the fact that these drugs may interfere with wound healing. Furthermore, temozolomide might cause neutropenia and lymphopenia and thus increase the probability of secondary infections^4^. Prophylactic measures include (1) optimal shaving, (2) removal of natural oils and any moisture and sweat prior to array placement, (3) regular array repositioning. Patients should be encouraged to wear breathable headwear to avoid overheating and inform nurses and caregivers about any changes in skin condition or skin sensation ^2,3^.

Topical applications for hyperhidrosis, xerosis, pruritus and contact dermatitis using aluminium chloride, corticosteroid solution/lotion or, also antihistamines might help to reduce pruritus. If symptoms persist, dermatologic consultation is recommended, furthermore, TTF should be interrupted, in the first instance for a few days up to a weak and then reassess.

Table S4. Recommendations for dose modifications of temozolomide (expert opinion)

| **Concomitant temozolomide with radiotherapy** | Dosing is 75 mg/m^2^daily throughout radiotherapy including weekends | |
| --- | --- | --- |
|  | Values | Action |
|  |  |  |
| Neutrophils | > 0.5 and < 1.5 x 10^9^/L | Delay temozolomide until normalization (neutrophils > 1.5 x 10^9^/L, platelets > 100 x 10^9^/L, CTC non-hematological toxicity grade < 1, except for alopecia, nausea and vomiting) |
|  | < 0.5 x 10^9^/L | Discontinue concomitant temozolomide |
|  |  |  |
| Platelets | > 10 and < 100 x 10^9^/L | Delay temozolomide until normalization (neutrophils > 1.5 x 10^9^/L, platelets > 100 x 10^9^/L, CTC non-hematological toxicity grade < 1, except for alopecia, nausea and vomiting) |
|  | < 10 x 10^9^/L | Discontinue concomitant temozolomide |
|  |  |  |
| Non-hematological toxicity (except for alopecia, nausea and vomiting) | CTCAE grade 2 | Delay temozolomide until the non-hematological toxicity recovers to grade < 1 (except for alopecia, nausea and vomiting) |
|  | CTCAE grade 3/4 | Discontinue concomitant temozolomide |
|  |  |  |
| **Maintenance temozolomide** | Dosing depends on severest toxicity during previous cycle, dose levels of temozolomide are 100 mg/m^2^, 150 mg/m^2^ and 200 mg/m^2^; dose re-escalation is not recommended.  Three dose level are defined^1^:  -1: 100 mg/m^2^ (dose reduction for prior toxicity at 150 mg/m^2^)  0: 150 mg/m^2^ (dose for cycle 1)  1: 200 mg/m^2^ (dose for cycle 2-6 in the absence of toxicity) | |
|  |  |  |
|  | Values | Action |
| Neutrophil nadir | < 1 x 10^9^/L | Reduce one dose level for next cycle |
| Platelet nadir | < 50 x 10^9^/L | Reduce one dose level for next cycle |
| Non-hematological toxicity (except for alopecia, nausea and vomiting) | CTCAE grade 3 | Reduce one dose level for next cycle  Discontinue if the same grade 3 non-hematological toxicity (except for alopecia, nausea, vomiting) recurs after dose reduction |
|  | CTCAE grade 4 | Discontinue maintenance temozolomide |
|  |  |  |
| Recurrent hematological toxicity requiring dose reduction below 100 mg/m^2^ temozolomide |  | Discontinue maintenance temozolomide |
| Recurrent CTCAE grade 3 non-hematological toxicity (except for alopecia, nausea and vomiting) |  | Discontinue maintenance temozolomide |

^1^The dose in cycle 1 (monotherapy) is 150 mg/m^2^ once daily for 5 days followed by 23 days without treatment. At the start of cycle 2, the dose is escalated to 200 mg/m^2^ if the CTC non-hematological toxicity for cycle 1 is grade ≤ 2, except for alopecia, nausea and vomiting, absolute neutrophil count is ≥ 1.5 x 10^9^/l, and the platelet count is ≥ 100 x 10^9^/l. If the dose was not escalated for cycle 2, escalation should not be done in subsequent cycles.

Table S5. Recommendations for dose modifications of lomustine^1^ (expert opinion)

| **Lomustine** | Dosing depends on severest toxicity during previous cycle. Dose levels of lomustine are 110 mg/m^2^, 90 mg/m^2^ and 70 mg/m^2^; dose re-escalation is not recommended. | |
| --- | --- | --- |
|  | Values | Action |
| Neutrophil nadir | < 1 x 10^9^/L | Reduce one dose level for next cycle |
| Platelet nadir | < 50 x 10^9^/L | Reduce one dose level for next cycle |
| CTCAE grade 3 non-hematological toxicity (except for alopecia, nausea and vomiting) |  | Wait until recovery to ≤ 1 and reduce one dose level for the next cycle |
| Recurrent CTCAE grade 3 non-hematological toxicity (except for alopecia, nausea and vomiting) despite dose reduction |  | Discontinue lomustine |
| CTCAE grade 4 non-hematological toxicity (except for alopecia, nausea and vomiting) |  | Discontinue lomustine |

Lomustine is given in 6-week cycles, with lomustine on day 1 commonly at 110 mg/m^2^, with dose reduction levels at 90 mg/m^2^ and 70 mg/m^2^. In many areas worldwide lomustine is only available in capsule or tablets of 40 mg, limiting the options for precise dosing. Rounding should go to the lower dose. In case of significant hematological toxicities during previous treatment with TMZ requiring dose reductions, a prudent approach with a reduced lomustine starting dose is indicated, with dose escalation in the absence of (hematological) toxicity during cycle 1. A cap of 200 mg regardless of body surface area is used in most institutions, with 40 mg dose reductions. The nadirs for hematological toxicity with lomustine are expected between days 28 and 35 and tend to worsen with more cycles. Routine diffuse capacity pulmonary function tests are required according to some regulatory approvals, but is unlikely to yield abnormalities or alter management. In case of respiratory complaints not otherwise accounted for pulmonary function tests can be considered. Routine anti-emetic prophylaxis one hour before lomustine intake is recommended, with either metoclopramide or (better) 5 HT3-antagonists.

The next cycle should be delayed until hematological recovery, (e.g., platelet counts over 100,000/µl and neutrophil counts over 1,500/µl) while monitoring hepatic and kidney function. In case of grade 3 or 4 toxicities a dose reduction by 25% or 40 mg is recommended, in case of significant grade 2 hematological toxicity and dose delays, one should anticipate more severe hematological toxicity in the next cycle.

Dose adaptations for myelosuppression during PCV polychemotherapy may differ across the globe, but some rules apply ^5,6^ vincristine needs no consideration, because it does not cause relevant myelosuppression. Some institutions base dose adaptations on the nadir, that is, nadirs prior to day 20-25 will result in dose reductions of procarbazine, usually ⅔, later nadirs will trigger dose reductions of lomustine. Other sites argue that lomustine is most likely to be the more active agent, notably in patients preexposed to TMZ and thus dose-reduce or stop procarbazine first before the dose of lomustine is reduced. If procarbazine is stopped because of other toxicity, mostly dermatological toxicity, most sites would also discontinue vincristine.

Table S6. Management of hematological toxicity

|  | **Thresholds*** | **Treatment** | **Prophylaxis** |
| --- | --- | --- | --- |
|  |  |  |  |
| Neutropenia | Absolute neutrophil count < 500/µL | Filgrastim, Lenograstim | Antibiotics |
|  |  |  |  |
| Lymphopenia | CD4 T cell count < 200/μL |  | Trimethoprin sulfamethoxazole and acyclovir |
|  |  |  |  |
| Thrombocytopenia | 10-20 x 10^3^/μL | Platelel transfusion |  |
|  |  |  |  |
| Anemia | Hemoglobin < 8 g/dL** | Packed red blood cell transfusion | Erythropoietin |
|  |  |  |  |

*can be lowered depending on age and comorbidities

**References**

1. Landriel Ibañez FA, Hem S, Ajler P, et al. A new classification of complications in neurosurgery. *World Neurosurg*. 2011;75(5-6):709-715; discussion 604-611. doi:10.1016/j.wneu.2010.11.010

2. Murphy J, Bowers ME, Barron L. Optune®: Practical Nursing Applications. *Clin J Oncol Nurs*. 2016;20(5 Suppl):S14-19. doi:10.1188/16.CJON.S1.14-19

3. Lacouture ME, Anadkat MJ, Ballo MT, et al. Prevention and Management of Dermatologic Adverse Events Associated With Tumor Treating Fields in Patients With Glioblastoma. *Front Oncol*. 2020;10:1045. doi:10.3389/fonc.2020.01045

4. Lukas RV, Ratermann KL, Wong ET, Villano JL. Skin toxicities associated with tumor treating fields: case based review. *J Neurooncol*. 2017;135(3):593-599. doi:10.1007/s11060-017-2612-8

5. Jutras G, Bélanger K, Letarte N, et al. Procarbazine, lomustine and vincristine toxicity in low-grade gliomas. *Curr Oncol*. 2018;25(1):e33-e39. doi:10.3747/co.25.3680

6. Esteyrie V, Dehais C, Martin E, et al. Radiotherapy Plus Procarbazine, Lomustine, and Vincristine Versus Radiotherapy Plus Temozolomide for IDH-Mutant Anaplastic Astrocytoma: A Retrospective Multicenter Analysis of the French POLA Cohort. *Oncologist*. 2021;26(5):e838-e846. doi:10.1002/onco.13701
